# Transitional B Cells and TLR9 Responses Are Defective in Selective IgA Deficiency

**DOI:** 10.3389/fimmu.2018.00909

**Published:** 2018-04-27

**Authors:** Andri L. Lemarquis, Helga K. Einarsdottir, Rakel N. Kristjansdottir, Ingileif Jonsdottir, Bjorn R. Ludviksson

**Affiliations:** ^1^Department of Immunology, Landspítali-University Hospital, Reykjavík, Iceland; ^2^Faculty of Medicine, University of Iceland, Reykjavík, Iceland; ^3^Division of Infectious and Inflammatory Diseases, deCODE Genetics, Reykjavík, Iceland

**Keywords:** selective IgA deficiency, B cells, T cells, IgA, CpG, T cell-independent B cell responses, Transitional B cells, B regulatory cells

## Abstract

Selective IgA deficiency (IgAD) is the most common primary antibody deficiency in the western world with affected individuals suffering from an increased burden of autoimmunity, atopic diseases and infections. It has been shown that IgAD B cells can be induced with germinal center mimicking reactions to produce IgA. However, IgA is the most prevalent antibody in mucosal sites, where antigen-independent responses are important. Much interest has recently focused on the role of TLR9 in both naïve and mature B cell differentiation into IgA secreting plasma cells. Here, we analyze the phenotype and function of T and B cells in individuals with IgAD following IgA-inducing CpG-TLR9 stimulations. The IgAD individuals had significantly lower numbers of transitional B cells (CD19^+^CD24^hi^CD38^hi^) and class-switched memory B cells (CD20^+^CD27^+^IgD^−^) *ex vivo*. However, proportions of T cell populations *ex vivo* as well as *in vitro* induced T effector cells and T regulatory cells were comparable to healthy controls. After CpG stimulation, the transitional B cell defect was further enhanced, especially within its B regulatory subset expressing IL-10. Finally, CpG stimulation failed to induce IgA production in IgAD individuals. Collectively, our results demonstrate a defect of the TLR9 responses in IgAD that leads to B cell dysregulation and decreased IgA production.

## Introduction

Selective IgA deficiency (IgAD) is the most common primary immune deficiency (PID), affecting about 1/700 individuals (1). It is characterized by IgA below <0.07 mg/dL with normal levels of IgG and IgM (2). IgA-deficient individuals are often asymptomatic, but are at an increased risk of diseases related to immune deregulation, autoimmune, and atopic diseases (3). The mechanisms leading to IgAD are most likely multifactorial involving several potential immunological pathways that are also common to the immunopathogenesis of autoimmunity, atopy, and infectious diseases (4). As has been the case with the elucidation of other PIDs, understanding the mechanisms leading to IgAD may deepen our understanding of the immune system, and could pave the way for a personalized approach to the diseases affecting these individuals (5). In addition, it is also well known that IgAD can develop into a more severe antibody deficiency phenotype such as common variable immunodeficiency or IgG deficiency, this is especially true for cases, where a *TNFRSF13B* coding variant is associated with the defect (6).

IgA is the most abundant antibody isotype produced in the body, and is secreted by terminally differentiated antibody secreting cells (ASC) (7). Although detected at a high concentration in blood, the most vital role of IgA is predominantly to interact locally with pathogens and antigens at mucosal surfaces (8). The mechanisms leading to the differentiation and survival of B cells to become ASCs are dictated by a variety of control mechanisms, including class switching, homing, co-stimulation, and finally commitment to a plasma cell lineage (7). Since the defect in IgA production in IgAD individuals could be due to a defect in any of these mechanisms it is important to delineate which pathways are defective as well as those functioning correctly in IgAD individuals.

Bone marrow transplantation in individuals with IgAD can cure the deficiency suggesting that the defect is of hematopoietic origin (9). A phenotypic analysis of peripheral blood (PB) lymphocytes in individuals suffering from IgAD has led to the prevailing view that defects in numbers and function of certain lymphocyte populations might be the main cause of IgA deficiency (10–12). Advances in multicolor flow cytometry and better biological understanding of B cell maturation have led to renewed interest in detailed phenotypic analysis of B cells and T cells in immune-mediated diseases. Some of the older studies about IgAD have shown lower numbers of switched memory B cells, classified as IgD-CD27^+^, and transitional B cells, classified as CD38^hi^IgM^+^ (12, 13) in adult donors. A more appropriate phenotypic definition of transitional B cells would be CD24^hi^CD38^hi^. A recent study found that this population to be within the normal range in pediatric IgAD individuals (14). It is of note that transitional B cells represent the majority of B cells in children and may, therefore, have a different function than in adults (14). Transitional B cells have not been studied so far in adult IgAD donors, and current knowledge on lymphocyte subpopulations could be greatly enhanced by recent advances in multicolor flow cytometry and better understanding of the biology of B cell maturation and differentiation. Transitional B cells have been shown to be important regulators of the immune system, in part through their IL-10 secretion and their B regulatory (Breg) phenotype assessed by IL-10 expression (15). They have been suggested to have a role in the pathogenesis of IgA-mediated diseases, such as IgA nephropathy (16), and have been implicated in the immunopathogenesis of autoimmunity (17).

The major mechanisms determining maturation stages during B-ontogeny from recently emerging B cells to terminally differentiated IgA^+^ plasma cells have not been fully clarified. However, cytokines, such as IL-10 and CD4^+^ T-regulatory cells (Tregs), are known to be important in this process. Accordingly, reduced Treg numbers have been reported in IgAD (18). IL-10 has furthermore repeatedly been shown to induce IgA production in IgAD B cells (19) with and without other cytokines such as IL-4 and IL-21 (20) in germinal center mimicking stimulations. However, these stimulations have not been shown to induce long-lived antibody responses. The induction of B-cell differentiation through the activation of innate immune pathways has been shown to be a strong inducer of IgA, presumably due to the key role of IgA in the innate and adaptive interactions in the gut where this isotype is most prevalent (21). No functional analysis exists of TLR9-driven B-cell activation in IgAD.

In this study, we evaluate the phenotype of PB T and B lymphocytes from IgAD individuals and the role of TLR9 in B cell maturation and IgA induction in IgAD.

## Results

### Demographics of Study Group

To analyze IgA deficiency, we harvested blood from deficient donors from the Icelandic IgAD cohort (3). The demographics and laboratory values of IgAD individuals and HC are shown in Table 1. No demographic differences were found between the two study groups. However, the IgAD group had significantly higher levels of total IgG with significantly higher IgG1 and higher autoantibody positivity as has previously been described (Table 1).

**Table 1:** Demographics of healthy controls (HC) and IgAD patients (IgAD).

	HC	IgAD		*p* Value
Age (mean ± SD years)	51(±12.5)	57(±11)		
M/F ratio	1	0.6		
IgM	1.0 ± 0.4	1.1 ± 0.4		
IgG	10.6 ± 3.1	14.8 ± 3.2	g/l	<0.0001
IgG1	7.1 ± 2.5	11.3 ± 3.5	g/l	<0.0001
IgG 2	3.4 ± 1.8	2.9 ± 1.2	g/l	
IgG3	0.4 ± 0.22	0.4 ± 0.3	g/l	
IgG4	0.4 ± 0.3	0.2 ± 0.2	g/l	
Autoantibody+	20%	53%		
Antinuclear antibodies (ANA+)	0%	40%		
Extractable nuclear antigens (ENA+)	0%	26%		
Rheumatoid factor (RF+)	13%	20%		
RF IgM+	0%	0%		
RF IgG+	0%	0%		
Cyclic citrulinated peptides (CCP+)	20%	33%		

*Table depicts age, male to female ratio (M/F ratio), prevalence of autoimmunity, IgM, IgG, and IgG subgroups, and the prevalence of autoantibodies (ANA, ENA, RF, and CCP) in the IgAD group examined. Statistical comparison of IgAD and HC was calculated using one-way ANOVA for IgM, IgG, IgG1, IgG2, IgG3, and IgG4*.

### IgAD Individuals Have Decreased Frequencies of Transitional B Cells

**Figure 1:**
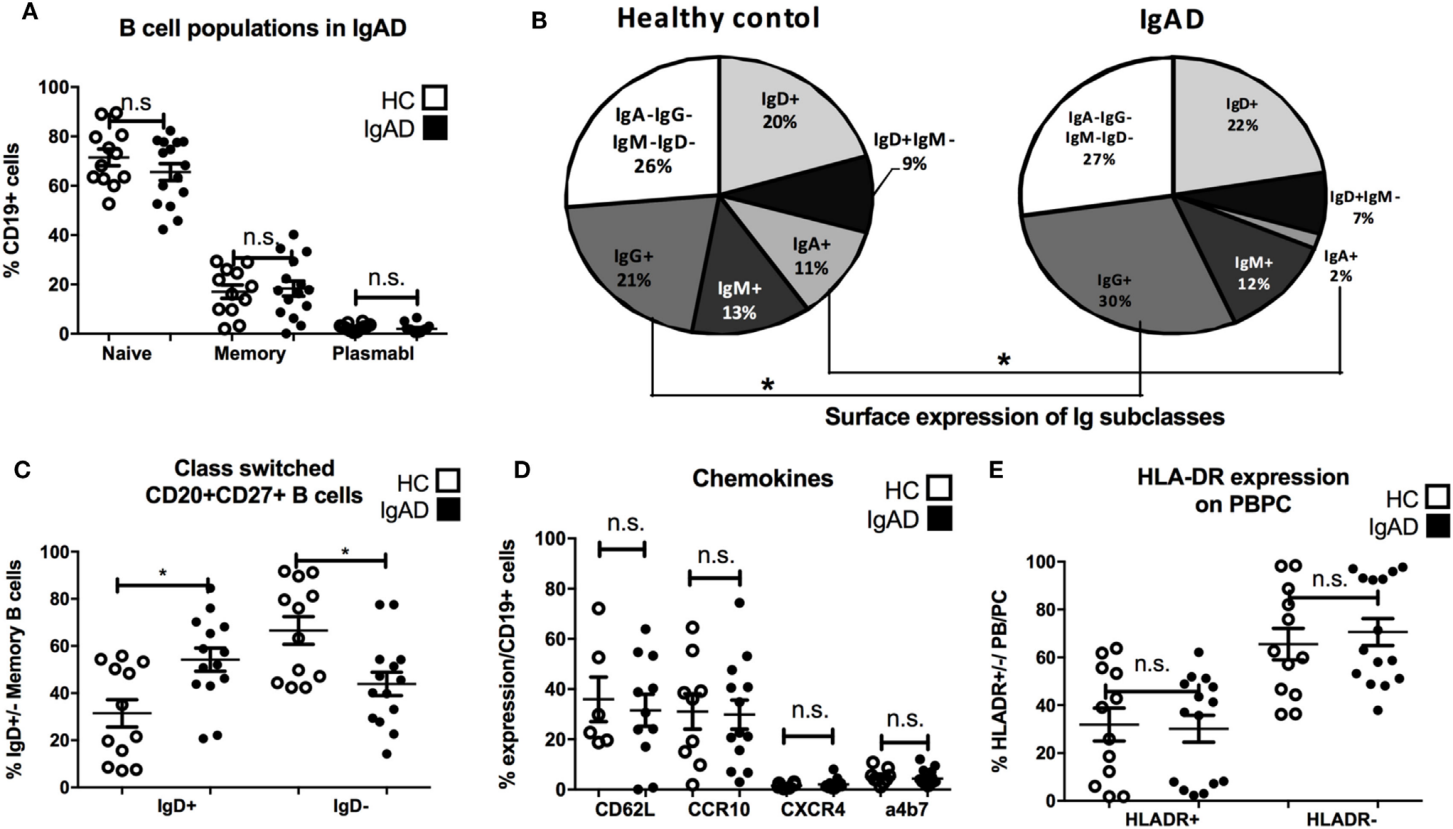
B cells in IgA-deficient individuals lack IgA, but have increased IgG surface expression. The figure shows B cell populations as measured by flow cytometry comparing selective IgA deficiency (IgAD) donors to healthy controls (HC). **(A)** Percentage of naïve (CD20^+^CD27^−^), memory (CD20^+^CD27^+^), and plasmablasts (CD20^−^CD27^+^) as % of CD19^+^ B cells are shown. No significant differences are seen for these populations between IgAD and HC. **(B)** Representative pie chart for frequencies of Ig isotypes expressed on total CD19^+^ B cells in IgAD and HC donors. The fraction is reduced for IgA (*p* < 0.0009) positive B cells and increased for IgG (*p* < 0.029) positive B cells in IgAD compared to HC. **(C)** The percentages of class-switched (CD20^+^CD27^+^IgD^+^) memory CD19^+^ B cells in IgAD individuals and HC. IgAD donors have significantly less class-switched memory B cells. **(D)** The proportion of CD19^+^ B cells expressing chemokines associated with gut homing; CD62L, α4β7, CCR10, and CXCR4, is comparable in IgAD individuals and HC. **(E)** The activation of plasmablasts/plasma cells in IgAD evaluated by percentage of HLA-DR expression shows comparable activation in IgAD and HC. All data are from five-independent experiments, no duplicates. Significance was calculated in relation to control group determined by one-way ANOVA. *p < 0.05, **p < 0.01, ***p < 0.001.

Through the use of multicolor flow cytometric analyses our understanding of the various lymphocyte sub-phenotypes and their different biology and function has been greatly enhanced. Thus, in order to elucidate this pattern for adult IgAD the following subsets were evaluated in the PB compartment of our IgAD study group and compared to HC. These include transitional (CD24^hi^/CD20^+^/CD38^hi^/CD27^−^), mature-naïve (CD20/CD27^−^), memory (CD20^+^/CD27^+^), and antibody secreting cells (CD20^−^/CD27^hi^). In addition, and to further delineate the phenotypic characteristics of these B cell subpopulations in IgAD, the expression of CD21, CD62L, CCR10, β7, CXCR4, kappa light chain, lambda light chain, IgM, IgG, IgD, and IgA was assessed as well. As expected the fraction of naïve, memory, and antibody secreting populations were found to be normal in IgAD (Figure 1A). The analysis of IgA, IgG, IgM, and IgD expression on total B cells demonstrates a defect in IgA expressing B cells (fivefold reduction compared to HC), together with significantly increased expression of IgG^+^ B cells within the IgAD group (Figure 1B). As could be expected and previously been shown by others (12) class-switched memory B cells (CD20^+^CD27^+^IgD^−^) were significantly decreased in our IgAD study group and non-class-switched B cells (CD20^+^CD27^+^IgD^+^) were similarly found to be increased (Figure 1C). The expression of the mucosal CXCR4, CCR10, and α4β7 homing receptors were also assessed in memory B cells with no difference observed between IgAD and HC (Figure 1D). The fraction of plasmablasts was normal in IgAD individuals indicating that even though a major part of B cells with a plasmablast phenotype (CD19^+^CD20^−^CD27^hi^) have been shown to be IgA^+^ in normal individuals, the increase in the IgG^+^ fraction compensates for this loss in IgAD (22). Finally, plasmablasts were found to be within normal range in the IgAD individuals, and not with an activation HLADR^+^ phenotype (Figure 1E).

### Transitional B Cells Producing IL-10 Are Decreased in IgAD Individuals

**Figure 2:**
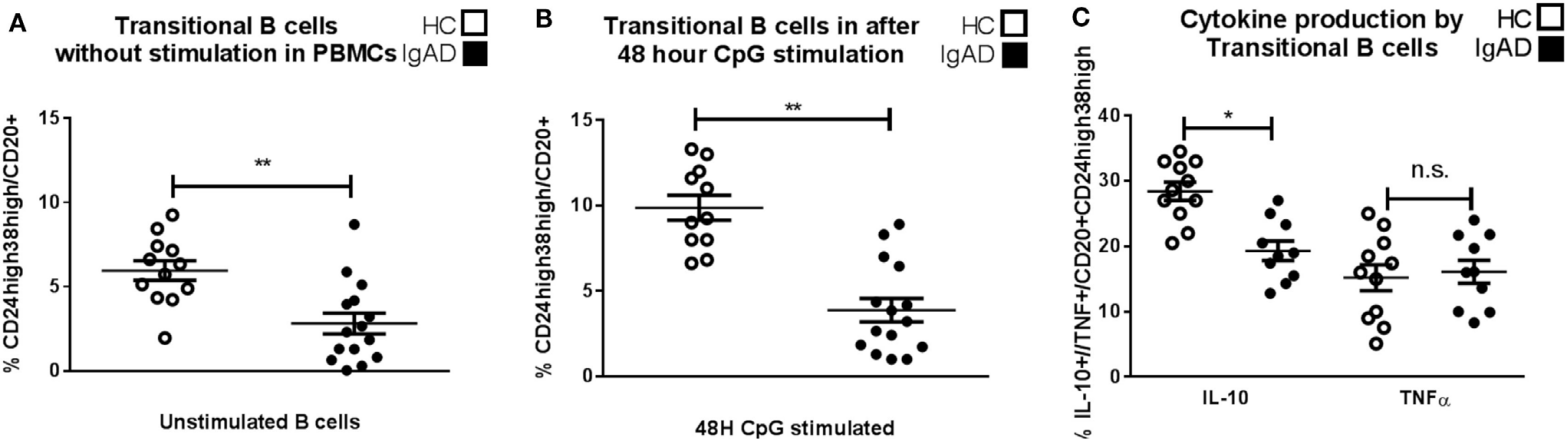
CD24^hi^CD38^hi^ transitional B cells in selective IgA deficiency (IgAD) individuals are defective in their numbers, inducibility, and IL-10-secreting properties. The figure shows transitional B cells as measured *ex vivo* in PBMCs and after *in vitro* stimulation of B cells by CpG for 48 h and regulatory B cells (Bregs) as measured by flow cytometry comparing IgAD and controls. **(A)** B cells with a transitional B cell phenotype (CD24^hi^CD38^hi^) are of lower fraction in IgAD individuals compared to healthy controls (HC) before stimulation and **(B)** after stimulation. Both IgAD and HC do though respond to CpG, as the fractions of the transitional population are increased after stimulation. **(C)** Evaluation of Breg function by the measurement of intracellular IL-10 demonstrate a lower fraction of IL-10^+^ Bregs (CD24^hi^CD38^hi^IL-10^+^) in IgAD compared to HC. A measurement of intracellular TNF-α in transitional B cells shows no difference between the two groups. In all figures, HC are depicted as white, while IgAD individuals are depicted as black bars. Data are shown as mean ± SEM of all IgAD and HC assessed. All data are from five-independent experiments, no duplicates. Significance was calculated in relation to the control group. *p < 0.05, **p < 0.01, ***p < 0.001 (one sided Student’s *t*-test).

To examine non-antibody dependent B cell responses in IgAD, transitional B cells were analyzed and found to be of lower proportion in the IgAD group compared to the HC, *p* < 0.02, Figure 2A. This was observed both in their frequency (Figure 2A) and total numbers (transitional B cell total numbers: IgAD; 5,649 ± 3,126 vs. HC; 2,183 ± 5,837; *p* < 0.02). In addition, their number did not correlate with the age of the IgAD individuals studied. Due to the observed transitional B cell deficiency in IgAD it was important to assess their functionality further. An emerging role for transitional B cells is their ability to be induced to produce IL-10 through TLR9 stimulation. Therefore, CD19^+^ B cells isolated from IgAD individuals were stimulated with CpG for 48 h prior to the addition of Brefeldin A, PMA, and ionomycin. As shown in Figure 2B, even under such strong inducing transitional B-cell differentiation conditions, this phenotype remained significantly reduced in the IgAD group. In addition, the fraction of IL-10^+^ expressing CD20^+^CD24^hi^CD38^hi^ transitional B cells *in vitro* was decreased in IgAD individuals (CD20^+^CD24^hi^CD38^hi^IL-10^+^), Figure 2C. This was not due to an overall decrease in cytokine production, since the fraction of TNFα^+^ transitional B cells was normal (CD20^+^CD24^hi^CD38^hi^TNFα^+^).

### Stimulation by CpG Fails to Normalize IgA Production in IgAD Individuals

**Figure 3:**
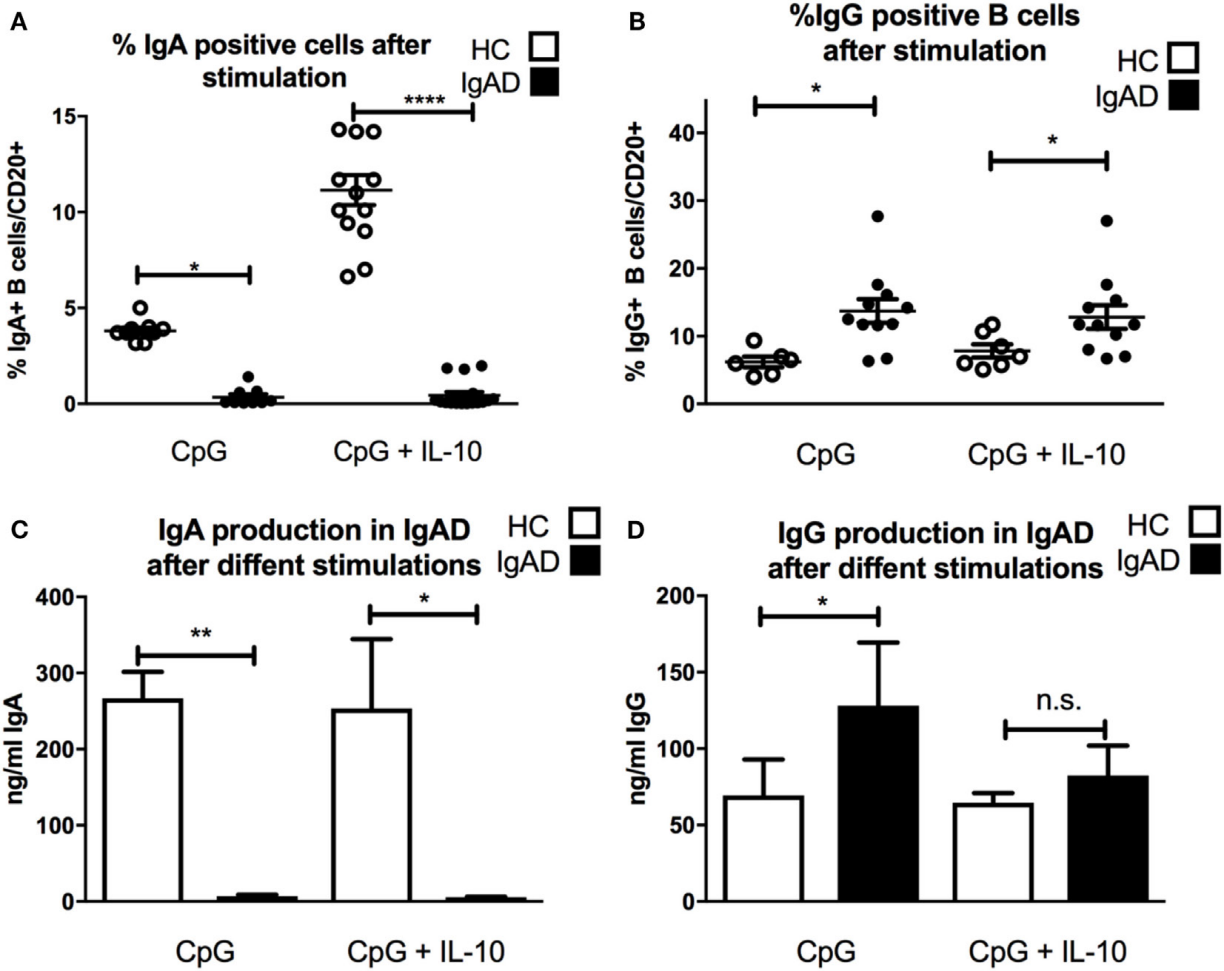
The lack of IgA surface expression and production is not corrected by TLR9 stimulation in selective IgA deficiency (IgAD) individuals. The figure shows the responsiveness of isolated CD19^+^ B cells in IgAD individuals and healthy controls (HC) after 7 days of CpG or CpG with IL-10 stimulation as evaluated by IgA and IgG expression and secretion evaluated by flow cytometry and enzyme-linked immunosorbent assay. IgAD donor B cells express a lower proportion of IgA **(A)** and high of IgG **(B)** following CpG stimulation. This unresponsiveness is not overcome by the addition of IL-10 **(A,B)**. In HC, an increase is seen in the proportion of IgA but not IgG positive B cells when IL-10 is added to the CpG stimulation **(A,B)**. IgA production **(C)** is seen following CpG stimulation in HC but not in IgAD individuals. Defective IgA secretion is not rescued in CpG stimulated IgAD B cells by the addition of IL-10. On the contrary, IgG production is significantly higher in IgAD individuals compared to HC after CpG stimulation **(D)**. This difference is less distinct after the addition of IL-10 and is not statistically significant. In all figures, HC are depicted as white, while IgAD individuals are depicted as black bars. Data are shown as mean ± SEM of all IgAD and HC assessed. All data are from five-independent experiments, no duplicates. Significance is shown as *p < 0.05, **p < 0.01, ***p < 0.001 (as determined by one-way ANOVA).

Since CpG-driven stimulation revealed a defective transitional B-cell differentiation and IL-10 expression investigated whether B cells from IgAD could be induced by CpG to produce IgA with the addition of exogenous IL-10. As could be expected CpG-induced B-cell stimulation led to a significant IgA secretion and surface expression in HC, however, this was not observed in IgAD individuals (Figures 3A,C). In addition, even under maximal stimulatory conditions including CpG with exogenous IL-10 administration this defect was not reverted (Figures 3A,C). Finally, this was further reflected by a higher production of IgG seen in IgAD individuals after CpG stimulation alone when compared to healthy controls. IgG surface expression and secretion remained higher in IgAD compared to HC after CpG-driven stimulation (Figures 3B,D). The same was seen for CPG^+^IL-10 stimulation for all, but IgG production where differences were not as big and failed to reach significance.

### IgAD Individuals Have a Normal Proportion and Function of Classical T-Cell Subpopulations

**Figure 4:**
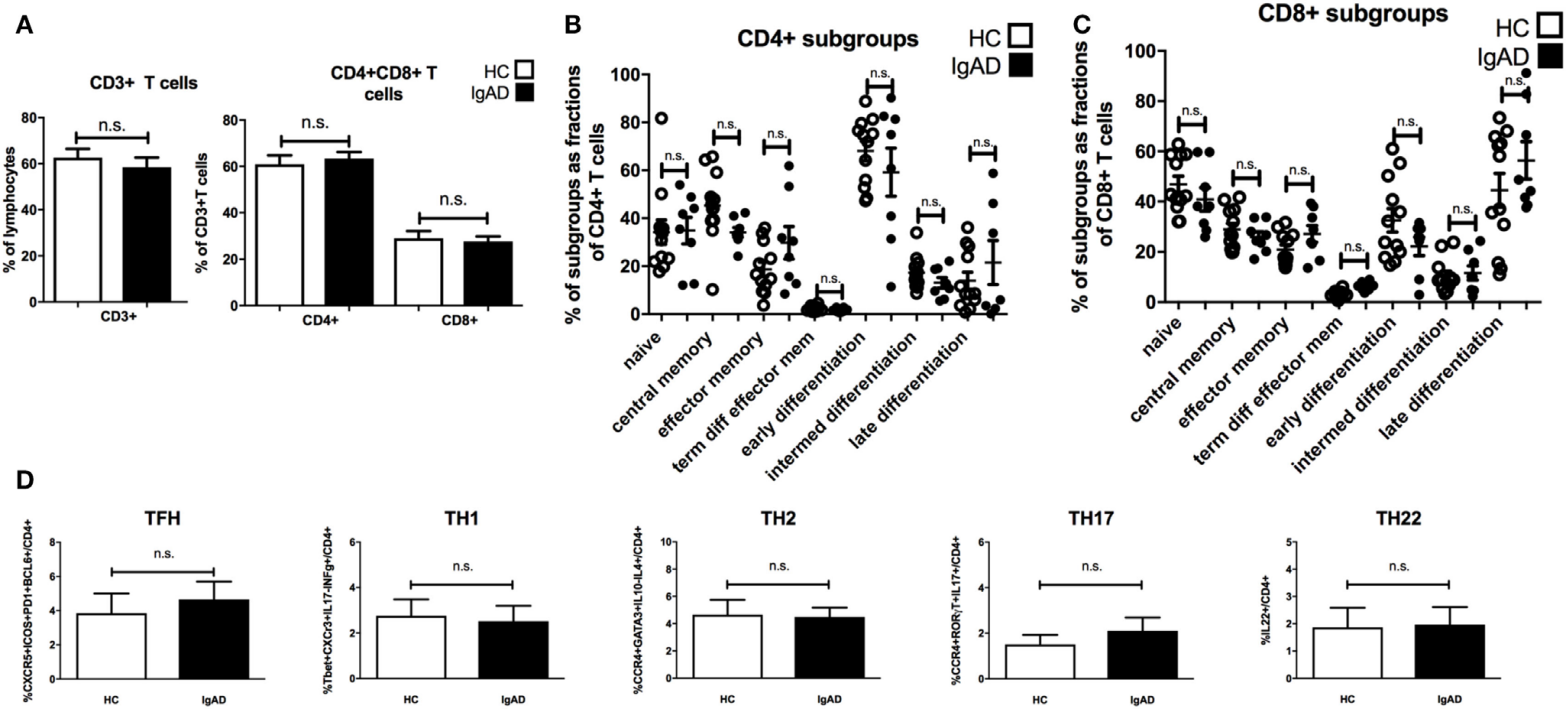
The main T cell subsets are of normal proportions in selective IgA deficiency (IgAD). The figure shows T cell populations as measured by flow cytometry on PBMCs for extracellular markers and on PBMCs stimulated with PMA and ionomycin for 4 h comparing IgAD and controls. No significant differences were found for the proportions of T cells. **(A)** The fraction of CD3^+^ T cell as proportions of lymphocytes and CD4^+^ and CD8^+^T cells as proportions of CD3^+^ T cells in IgAD vs. healthy controls (HC). **(B)** Fractions of the different T cell subpopulations of CD4^+^ and **(C)** CD8^+^ T cells. The subpopulation gating is based on their classification as naïve (CD62L^+^CCR7^+^), central memory (CD62L^−^CCR7^+^), effector memory (CD62L^−^CCR7^−^), terminally differentiated effector memory (CD62L^+^CCR7^−^), early differentiated (CD27^+^CD28^+^), intermediately differentiated (CD27^+^CD28^−^), or lately differentiated (CD27^−^CD28^−^). **(D)** The figures show the proportions of T helper cell populations as a fraction of CD4 T cells. No significant differences were seen between T follicular helper cell [CD4^+^CXCR5^+^ICOS^+^ programmed cell death protein 1 (PD1^+^) B-cell lymphoma 6 protein^+^], TH1 (CD4^+^T-box transcription factor TBX21^+^CXCR3^+^IL-17^−^IFNγ^+^), TH2 [CD4^+^CCR4^+^trans-acting T-cell-specific transcription factor (GATA3^+^) IL-10^−^IL-4^+^], TH17 (CD4^+^CCR4^+^RORγT^+^IL-17^+^), and TH22 (CD4^+^IL-22^+^) cells between IgAD and healthy controls. All data are from five-independent experiments, no duplicates. Significance was calculated in relation to the control group. *p < 0.05, **p < 0.01, ***p < 0.001 (as determined by one-way ANOVA).

Antibody production can be heavily influenced by T cell signaling with subpopulations of T cells influencing different isotype switching as well as being important in germinal center B cell maturation. Therefore, a detailed phenotypic analysis of the various subpopulations of T-cells was performed. Our results suggest that proportions of CD3^+^, CD4^+^, and CD8^+^T cells are comparable in IgAD individuals compared to healthy donors (Figure 4A). Additionally, no differences were seen between the two groups for any of the measured CD4^+^ (Figure 4B) and CD8^+^(Figure 4C) T-cell subpopulations. The expression of surface markers CD27, CD28, CD62L, and CCR7 was used to define the following T cell subpopulations (flow cytometry gating depicted in Figure S1C in Supplementary Material); naïve (CD62L^+^CCR7^+^), central memory (CD62L^−^CCR7^+^), effector memory (CD62L^−^CCR7^−^), terminally differentiated effector memory (CD62L^+^CCR7^−^), early differentiated (CD27^+^CD28^+^), intermediately differentiated (CD27^+^CD28^−^), or late differentiated (CD27^−^CD28^−^) T cells. T-effector functions were further assessed by the stimulation of PBMCs with PMA and ionomycin in the presence of brefeldin A followed by staining for detection of intracellular cytokines and transcription factors. Such stimulation did not reveal any significant difference of the various effector T-cell subpopulations between IgAD and HC in the following T-effector cell types (Figure 4D): T follicular helper cell (TFH) (CD4^+^CXCR5^+^ICOS^+^ programmed cell death protein 1 (PD1^+^) B-cell lymphoma 6 protein (BCL6^+^), TH1 (CD4^+^ T-box transcription factor TBX21^+^CXCR3^+^IL-17^−^IFNγ^+^), TH2 [CD4^+^CCR4^+^trans-acting T-cell-specific transcription factor(GATA3^+^)IL-10^−^IL-4^+^], TH17 (CD4^+^CCR4^+^RORγT^+^IL-17^+^), and TH22 (CD4^+^IL-22^+^).

### Natural and Induced Tregs Have Normal Numbers and Function in IgAD

**Figure 5:**
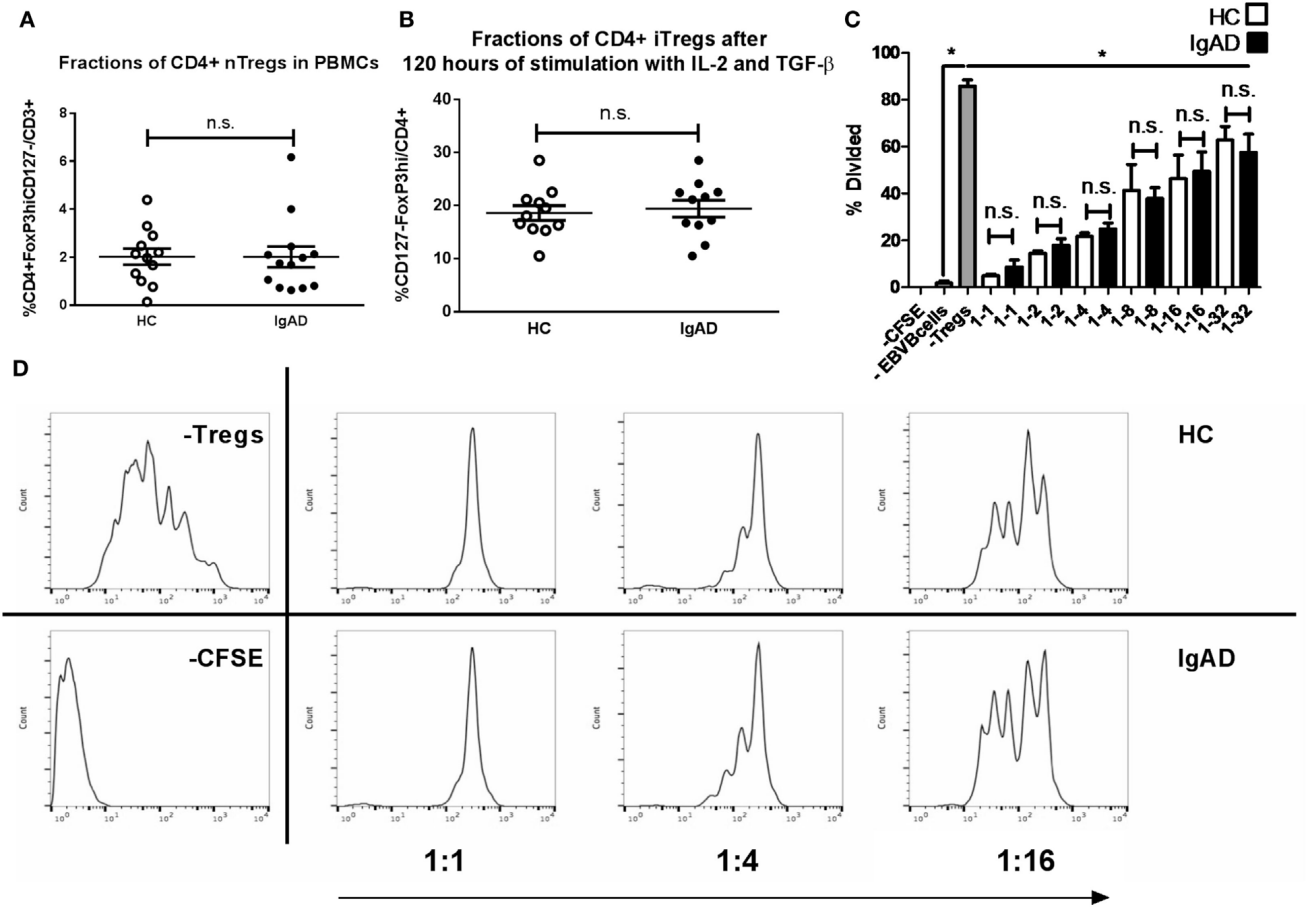
The numbers, inducibility, and functions of Tregs are not hampered in selective IgA deficiency (IgAD). The figure shows natural Tregs (nTregs) as measured by flow cytometry on *ex vivo* PBMCs **(A)**, induced Tregs (iTregs) on CD4^+^CD25^−^ T cells stimulated for 4 days by anti-CD3ε, anti-CD28, IL-2, and transforming growth factor beta 1 **(B)**, and suppressive capacity of the iTregs as measured by a suppression assay with super antigen-pulsated Epstein–Barr transformed B cells (EBV-B) cells and allogenic PBMCs **(C)**, comparing IgAD and controls. **(A)** The proportion of cells expressing a CD4 nTreg phenotype characterized by CD4^+^CD127 ^−^CD25^hi^forkhead boxP3(FoxP3^hi^) expression was not found to differ in IgAD from healthy controls (HC). **(B)** Neither did *in vitro* induced CD4^+^ iTregs after 4 days of stimulation. **(C)** The suppressive capacity of the iTregs from IgAD is intact and comparable to HC at different ratios when comparing proliferation of fluorescent 5,6-carboxyfluorescein succinimidyl ester (CFSE) stained CD8 T cells in allogeneic co-cultured PBMCs stimulated by super antigen-pulsated EBV-B cells. **(D)** A representative experiment showing a HC and an IgAD individual in a suppressive assay, −CFSE is a negative control with no staining, −Tregs shows a positive control of proliferation without Tregs. All data are from five-independent experiments, no duplicates. Significance was calculated in relation to the control group. *p < 0.05, **p < 0.01, ***p < 0,001 (as determined by one-way ANOVA).

Previous studies have shown an interaction between IL-10^+^ Bregs and Tregs. Since Bregs were shown to be abrogated in IgAD, a phenotypic analysis of Tregs was undertaken. To fully understand the role of Tregs in the pathophysiology of diseases and defects one needs to look both at the natural Tregs (nTregs), induced Tregs (iTregs), and their function. nTregs were measured in PBMCs stimulated *in vitro* for 4 h with PMA and ionomycin and in the presence of brefeldin A (Figure 5A). iTregs were induced from isolated CD4^+^CD25^−^ T cells by stimulating them with anti-CD28, IL-2, and transforming growth factor beta 1 (TGF-β1) on anti-CD3ε-coated plates. No differences were seen between IgAD individuals and HCs for either population (Figures 5A,B). We further assessed the functional capacity of these iTregs in a previously published model using proliferation of fluorescent 5,6-carboxyfluorescein succinimidyl ester (CFSE) stained CD8^+^ T cells (from HC) in a co-culture with iTregs (from IgAD or HC) and super antigen-pulsed Epstein–Barr transformed B cells (EBV-B) cells. The iTregs from IgAD individuals showed normal suppressive capacity compared to iTregs from HC at the different dilutions of iTregs: EBV from 1:1 to 1:32 (Figures 5C,D).

### IL-10 Fails to Act as a Mediator of B-Cell Longevity in IgAD Individuals

**Figure 6:**
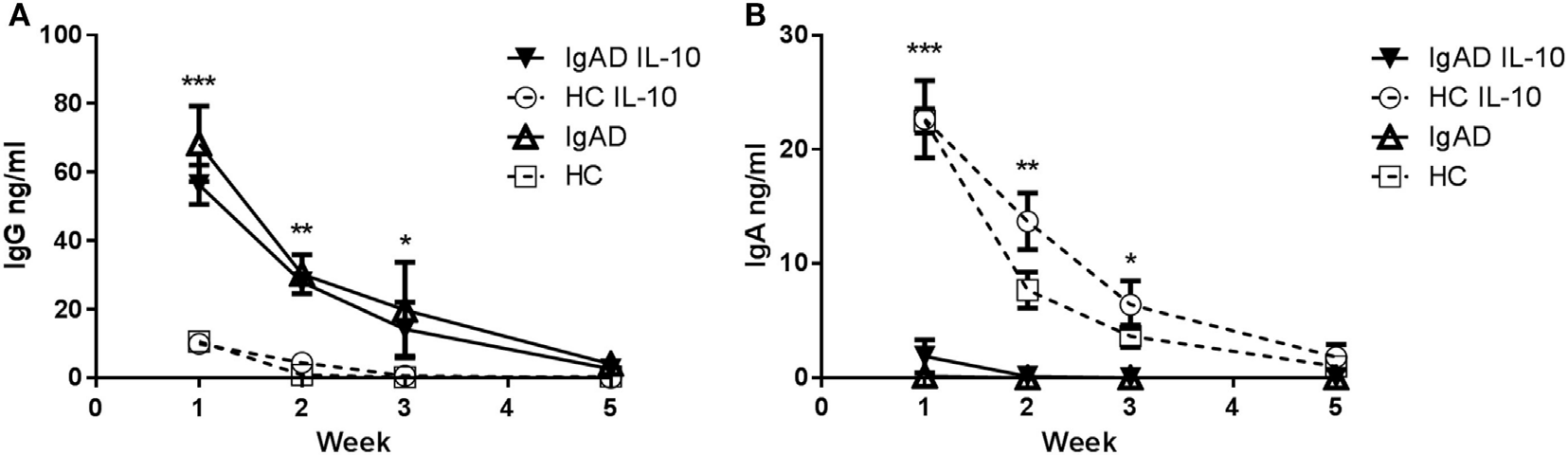
The IgA production induced by IL-10 does not result in a long-lived response in selective IgA deficiency (IgAD) individuals. The figure shows the antibody response of B cells from IgAD and healthy controls (HC) PBMCs with or without IL-10 stimulation as measured by enzyme-linked immunosorbent assay. **(A)** IgG production is statistically higher in IgAD individuals compared to HC at week 1 with production seen up to 5 weeks. **(B)** A small amount of IgA production is seen at week 1 in PBMCs from IgAD individuals with the exogenous addition of IL-10 (with—▾/or without IL-10 Δ), it remains significantly lower at 1 week compared to HC (with—○/or without IL-10 □), with production seen up to the fifth week in HC but not IgAD. Significance was calculated in relation to the control group. *p < 0.05, **p < 0.01, ***p < 0.001 (as determined by one-way ANOVA).

IL-10 has previously been shown to induce a short-lived increase in IgA production in IgAD (19). Our findings showed that Bregs secreting IL-10 are defective after TLR9 stimulation in IgAD, prompted us to explore this further especially, since exogenous cytokine stimulation has been proposed as treatment in PIDs such as IgAD and CVID (20). In a model mimicking a long-lived *in vivo* mucosal IgA [previously described in Ref. (23)], we explored whether B cells cultured together with other PBMCs and exogenous IL-10 could be forced into class switching and sustaining long-lived *ex vivo* IgA production. Robust IgA levels were detected in healthy donors for 3–5 weeks following stimulation, but only weak IgA levels were detected in IgAD donor cultures, and no longer detected after 2 weeks, indicating only short-lived responses following stimulation in IgAD (Figure 6A). The opposite was found for IgG production in IgAD, where a long-lived response was seen for up to 3 weeks and with a significantly higher production on week 2 following IL-10 stimulation (Figure 6B). The difference between the two groups of donors reached significance for the first 3 weeks for conditions with and without IL-10. This model demonstrated that IL-10 stimulation in the presence of other immune cells induces only a modest, short-lived IgA production in IgAD individuals, but leads instead to robust IgG responses, in stark contrast to the response observed in all HC.

## Discussion

In this study, we identify a defect in the cellular phenotype of IgAD B cells and their responses to TLR9 stimulation. The defect in PB is present as previously shown as a reduction in class-switched B cells (CD20^+^CD27^+^IgD^+^) but also in their precursors; CD19^+^CD24^hi^CD38^hi^ transitional B cells. After CpG stimulation of PBMCs IgA production remained defective in IgA-deficient individuals and they had significantly fewer IL-10^+^ transitional Breg cells compared to HC.

It has been demonstrated that antibody memory may not only be due to antigenic recall, but also due to nonspecific recall through interactions between components of the innate immune system and B cells. The effect of TLR9 stimulation has been repeatedly shown to induce antigenic responses both from memory and naïve B cells, pointing toward a mechanism where both antigenic recall and nonspecific recall may coexist (24). The stimulation through TLR9 in B cells has furthermore been shown to preferentially induce the IgA isotype, and more efficiently than other more conventional stimulations with CD40 ligand, anti-Ig, and cytokines (14). In IgA deficiency, certain germinal center mimicking antigen-dependent stimulations have been shown to induce IgA production while nothing is known about the effects of TLR stimulations on IgA production in IgAD.

TLR9 stimulation has been shown to prolong the lifespan of mature-naïve B cells *in vitro* (24). This has been hypothesized to maintain the normal B cell compartment. However, this proposed mechanism is challenged for IgA-producing B cells by normal numbers of CD19^+^ B cells found in our study. In contrast with our findings in adults, children with IgAD have been recently found to have normal numbers of transitional B cells numbers. In that same publication, the transitional B cell compartment is shown to be very different compared to adults (14). This could coincide with the fact that immune dysregulation develops at an increasing age in IgAD. We do not think the observed TLR9 defect in IgAD cells reflects a geronto immunological mechanism (23) since our IgAD group contained had only 2 individuals out of 15 above the age of 65 and only 1 in the HC, with no correlation observed between transitional cell number and age (Figure S2B in Supplementary Material).

Regulatory B cells (Bregs, CD24^hi^CD38^hi^IL-10^+^) have been of great interest in recent years and are known to be inducible by TLR9 ligand CpG (17). In this study, we evaluated IL-10 expression in TLR9-stimulated B cells. IgAD individuals were found to have significantly lower numbers of IL-10^+^CD24^hi^CD38^hi^ Bregs after *in vitro* CpG stimulation.

Our results are on the whole mostly in agreement with what has been described in the literature, with the exception that we do not observe T cell defects in the main CD4 and CD8 populations, nor in Tregs. In contrast, Tregs have been shown to be of decreased proportions in a subgroup of IgAD individuals with chronic inflammation (18). Our observations are of interest, since Bregs are known to induce differentiation of Tregs, which are themselves a powerful inducer of IgA production. The previous reported differences in Tregs of IgAD donors did not contain our strict Tregs definition strategy (25). Our gating using CD4^+^CD25^hi^CD127^−^forkhead box P3(FoxP3^hi^) Tregs in IgAD showed both normal numbers of nTregs and iTregs with normal suppressive function in an induced inflammation model. This is important, since the sole use of nTregs in groups prone to inflammation, and immune dysregulation could be misleading and has been shown to be greatly enhanced by functional assessment (26). Due to the difficulties involved in recruiting rare donors from a small population such as Iceland, the IgAD cohort consisted of only 15 participants. The limited number of subjects included in the study could possibly have caused us to miss some subtle differences in cellular variations possibly detectable in a bigger cohort, which could have been sub-grouped with regards to clinical symptoms displayed by the participants.

The absence of IgA production observed following CpG stimulation demonstrates a defective T cell-independent response of B cells in IgA deficiency as opposed to prior findings indicating a defective T cell-dependent inducibility of IgA. However, the IL-10 defect seen following CpG is in concordance with these previous findings (19, 20). These previous studies showing short-lived IgA responses in IgAD following IL-10, taken together with the IL-10 defect we observed in Bregs, made us reason that naïve or memory B cells from IgAD donors stimulated with IL-10 or IL-10 inducing stimulations in their most physiological environment might mature to become long-lived IgA producing plasma cells. To evaluate this, PBMCs were stimulated and repeatedly evaluated for their IgA and IgG production. As expected, IL-10 did induce a very strong- and long-lived response in HC. However, it failed to do so in IgAD individuals, inducing only a weak surge in the first week (Figure 5A).

Prior results have repeatedly described the rescue of IgA production in IgAD with germinal center mimicking stimulations (19, 20, 27), while our model, which in contrast mimicks the TLR9-driven gut innate—adaptive immune interactions, was unable to correct the IgA defect. Our findings demonstrate a novel TLR9-mediated defect in IgAD at the interface of the innate and adaptive immune system.

TLR9 stimulation is one of the strongest IgA inducers and the IgAD defect is much more pronounced in gut-mimicking conditions than that which is seen for T cell-dependent stimulations (Figures S2C,D in Supplementary Material). Interestingly, IgG was seen at higher concentration in IgAD individuals compared to HC after this *in vitro* stimulation, reflecting the respective concentrations in serum. Although others have witnessed the same phenomenon, to our knowledge no reports have demonstrated a mechanism for this concomitant IgG increase, which is not observed in other hypogammaglobinemias. We do not believe this to be an effect of bypassing faulty IgA production at the level of gene class-switch recombination, since constant regions gamma 1 and gamma 3, encoding for isotypes IgG1 and IgG3 which are both increased in IgAD (28), precede the first alpha chain (encoding for IgA1). This fact points toward a mechanism related to biological preference *via* signaling instead of practical bypassing during genetic recombination in the B cell. It is intriguing that although potent T cell-dependent stimulations have shown to be able to nearly normalize IgA production in IgAD (19, 20) in our hands, the T cell subpopulations which are important in inducing class switch recombination are normal, both with regards to serum proportions and cytokine and transcription factor expression. We, therefore, hypothesize the defect to be on the B cell side, at the early differentiation stage of B cells close to their emergence from the bone marrow and in their TLR9 stimulation pathway.

Finally, our observations with respect to TLR responses may be relevant to normal and pathological immune responses beyond IgA deficiency. For example, transitional B cell defects and TLR defects have been seen in other PIDs, autoimmune infectious, and atopic diseases and can be affected by novel biologics used for the treatment of autoimmunity. These observations in combinations with a better insight into the underlying defect responsible for IgAD could lead to a deeper understanding of the responsiveness of innate signaling in B cells and its importance for immune homeostasis. However, a more comprehensive analysis of the transcriptomic changes occurring in B cells after TLR stimulation would be required to elucidate the biological processes and pathways involved.

## Materials and Methods

### Study Population

Peripheral blood from 15 IgA-deficient individuals (median age 51, range 39–76 years) with known IgAD from the Icelandic IgAD group were collected into heparinized tubes after obtaining informed consent (in accordance with procedures approved by The National Bioethics Committee and The Data Protection Authority in Iceland). These were compared to gender and age matched HC (median age 57, range 39–79 years).

### Isolation of PBMCs, B, and T Cells

Peripheral blood mononuclear cells (PBMCs) were isolated from heparinized peripheral blood using Ficoll–Paque gradient centrifugation (Sigma-Aldrich, St. Louis, MO, USA). CD19^+^ B cells were isolated by magnetic bead-based positive selection using Dynabeads and DETACHaBEAD CD19 (Invitrogen). CD4^+^ T cells were isolated with Dynabeads CD4 (invitrogen) and CD25^+^ T cells were depleted from the CD4^+^ T cell population using Dynabeads CD25 (invitrogen). All isolations were done according to the manufacturer’s instructions. The purity of isolated CD19^+^ B cells was consistently >95% as analyzed by flow cytometry.

### Flow Cytometric Analysis

For extracellular staining single-cell suspensions were prepared and surface molecules were stained for 20 min at 4°C with a stain for viable cells using LIVE/DEAD fixable Yellow stain kit (Invitrogen) and optimal dilutions of antibodies for the following anti-human mAb: CD3 PE-Cy7 (UCHT1), CD3 PerCP-Cy5.5 (OKT3), CD4 eFluor 450 (RPA-T4), CD8a APC-eFluor 780 (RPA-T8), CD19 PerCP-Cy5.5 (HIB19), CD20 APC-eFluor 780 (2H7), CD24 PE-Cy7 (eBioSN3), CD25 PE (BC96), CD27 eFluor 450 (eBioSN3), CD38 PE-eFluor 610 (HIT2), CD45RO PE (UCHL1), CD45RA (JS-83), CD127 FITC (eBioRDR5), CD194 (CCR4), PerCP-eFluor 710 (D8SEE), CD278 (ICOS) APC (ISA-3), CD279 PD-1 (EH12.2H7), IgM FITC (SA-DA4), HLA-DR eFluor 450 (L243), CCR10 PE (6588-5), CCR7 (3D12), β7 (FIB504), CCR4 (D8SEE), and CD185 (CXCR5) APC (MU5UBEE) all from affymetrix eBioscience; CD3 Brilliant Violet 510 (OKT3), CD14 Brilliant Violet 510 (M5E2), CD62L FITC (DREG-56), IgD Alexa Fluor 700 (IA6-2), CXCR4 (12G5) all from Biolegend, IgG PE (JDC-10) from abcam, and IgA APC (IS118E10) from Miltenyi-Biotec.

For intracellular staining single-cell suspensions were prepared and surface molecules were stained as described above. They were subsequently fixed with paraformaldehyde and permeabilized with either 0.5% saponine or FoxP3/transcription factor staining set (catalog nr 00-5523-00, eBio-sciences, San Diego, CA, USA), stained according to the manufacturer’s instructions for the following anti-human mAb: FoxP3 APC (236A/E7), T-bet PerCP-Cy5.5 (eBio4B10), BCL-6 PerCP-eFluor 710 (BCL-UP), GATA3 eFluor 660 (TWAJ), TNF alpha PE (MAb11), IL-4 PE (8D4-8), IL-6 Alexa Fluor 700 (MQ2-13A5), IL-10 Alexa Fluor 488 (JES3-9D7), IL-17 PE (eBio64CAP17), and IL-22 PE-Cy7 (22URTI) all from affymetrix eBioscience, IFNγ APC (4S.B3) from Biolegend. To assess proliferation fluorescent 5,6-carboxyfluorescein succinimidyl ester (Life Technologies) labeling was used according to manufacturer’s instructions. Stained cells were subsequently analyzed using 10 colors Beckman Coulter Navios Flow Cytometer.

### B Cell Culture Conditions

Isolated CD19^+^ B cells were cultured in Iscove’s modified Dulbecco’s medium (IMDM) supplemented with 10% heat-inactivated fetal calf serum (FCS), penicillin/streptomycin (100 U/ml), and 2 mM Glutamax in 96-well round bottom plates at a density of 50 × 10^4^ cells/well. T cell-independent mimicking stimulations were performed in 96-well round bottom plates in the presence of CpG-ODN2006 (1 µg/ml TLR9 agonist, InvivoGen). T cell-dependent mimicking stimulations were performed in 96-well round bottom plates with 0.5 µg/ml mouse anti-human-CD40 antibody (Ebioscience #16-0409-85), 5 µg/ml goat anti-human-IgM antibody (Jackson ImmunoResearch Laboratories, West Grove, PA, USA) with or without the presence of IL-10 (Invitrogen, 50 ng/ml). Assessment of the induced transitional CD24^hi^CD38^hi^ phenotype and Bregs was done on B cells harvested after 48 H CpG (1 µg/ml) stimulation. They were further blocked with brefeldin A for 6 h and re-stimulated with PMA and ionomycin for 4 h for the assessment of cytokine producing B cells by intracellular flow cytometry. For the assessment of TLR9 responsiveness of B cells they were cultured further and assessed for surface expression of antibody isotypes and production of immunoglobulins after 7 days. Supernatants were harvested and cells stained for flow cytometric analysis.

### A Model for the Assessment of the Spontaneous Production of IgA by B Cells

To assess spontaneous IgA production in IgAD after stimulation with IL-10, PBMCs were cultured at a density of 2 × 10^5^ cells/well in IMDM supplemented with 10% heat-inactivated FCS, penicillin/streptomycin (100 U/ml), and 2 mM Glutamax with and without the exogenous addition of IL-10 as previously described (29). Supernatants were collected at day 7, 14, 21, and 34 and replaced each time with the same amount of medium without additional stimuli. The collected supernatants were further measured by enzyme-linked immunosorbent assay (ELISA) to assess IgA and IgG production.

### T Cell Culture Conditions

T helper cell subsets were assessed by intracellular flow cytometry on PBMCs stimulated in 96-well round bottom plates with PMA and ionomycin for 4 h. T regulatory cell induction was done by culturing CD4^+^CD25^−^ isolated T cells in serum-free medium (Aim V, Invitrogen) in 96-well round bottomed cell culture plates with plate bound anti-CD3ε (UCHT) with soluble anti-CD28 for 120 h with IL-2 (100 IU/ml) and TGF-β1 (10 ng/ml) (antibodies and cytokines used are from R&D Systems, Abingdon, UK) for 5 days and then harvested to measure their maturation as induced Tregs (iTregs) by FACS. Their suppressive capacity was assessed as previously described (30) after harvest by co-culturing Tregs with CFSE labeled allogeneic PBMC’s and super antigen-pulsated Epstein–Barr transformed B cells (EBV-B cells) in Aim V serum-free medium (Invitrogen). Staphylococcal enterotoxins (SEA, SEB, and SEE, Toxin Technologies, 1 µg/ml of each) were used for 2 h pulses before washing three times with PBS. The PBMCs: EBV-B cells and iTregs: EBV-B cells ratio was constant at 10:1 while the iTregs: PBMCs ratio varied from 1:1 to 1:32. The cells were harvested after 3 days and the suppressive capacities of iTregs assessed by flow cytometric analysis by proliferation calculations.

### Measurement of IgG and IgA by ELISA

IgG, IgA, and IgM measurements were done with a standard kit from Bethyl Laboratories for the quantification of production in culture supernatants according to the manufacturer’s instructions as previously described (Bethyl Laboratories, E88-102 and E88-104) on 96 well plates (Nunc maxisorp plates, Denmark).

### Measurement of Autoantibodies

For the determination of cyclic citrulinated peptides in human sera, the Immunoscan CCplus^®^ test kit (Euro Diagnostica AB, Lundavägen 151, SE-212 24 Malmö, Sweden) was used. The screening for rheumatoid factor (RF) was performed using ELISA technique (Anti-Human Kappa Light Chain, Sigma). Antinuclear antibodies were assessed by indirect immunofluorescence (IIF) using rat tissue (ANA) is a screening for non-organ-specific antibodies.

### Flow Cytometric Analysis

Proliferation calculations were done using Flow Jo Tree star V7.6.5. Expression of cell surface markers was measured by flow cytometry and analyzed using Flow Jo Tree star VX.0.7. Basis of gates was determined with the use of fluorescence minus one (FMO) together with appropriate isotype controls. Results are presented as average with SEM.

### Statistical Analysis

Statistical analyses and graphs were made using GraphPad Prism (V 5.04 GraphPadSoftware Inc., for windows, La Jolla, CA, USA). Normality of the data was assessed using Kolmogorov–Smirnov test. Majority of obtained values were different from normal, therefore, the nonparametric Mann–Whitney *U*-test was performed. Results are expressed as mean ± SEM. Probability values <0.05 were considered significant.

## Ethics Statement

This study was carried out in accordance with the recommendations of The National Bioethics Committee and The Data Protection Authority in Iceland with written informed consent from all subjects. All subjects gave written informed consent in accordance with the Declaration of Helsinki. The protocol was approved by The National Bioethics Committee and The Data Protection Authority in Iceland.

## Author Contributions

AL, HE, and BL conceived and designed the experiments. AL and RK performed the experiments. IJ contributed reagents, materials and analysis tools. AL, HE, and BL wrote the paper.

## Conflict of Interest Statement

The authors declare that the research was conducted in the absence of any commercial or financial relationships that could be construed as a potential conflict of interest.

## Supplementary Material

Figure S1 Gating hierarchy to identify subsets of T and B lymphocytes. The figure shows the gating strategy described in the methods for T and B cells. **(A)** Cells were first identified based on forward scatter and side scatter and doublets excluded by forward area and forward height properties. **(B)** B cells were identified based on their expression of CD19, while excluding CD14^+^ and CD3^+^ positive cells and dead cells. Then a gating was done based on CD19 and CD27 to get CD19^lo^CD27^hi^ B cells. B cells were further analyzed based on their different expression of CD20 and CD27 for naïve, memory, and plasmablasts/plasma cells; CD24 and CD38 for transitional B cells, IgD, and CD27 for class-switched memory B cells. A gating is shown for HLADR expression on plasmablasts/plasma cells and for the expression of IgA and IgG in both healthy controls (HC) and IgAD individuals on plasmablasts/plasma cells. **(C)** T cells were identified based on their expression of CD3 and furthermore evaluated for their expression of CD4 and CD8. The subsets were subsequently analyzed for their expression of CD28 vs. CD27 and CCD62L vs. CCR7. Natural Tregs and induced Tregs cells were assessed based on their expression of CD4, CD127 negativity, CD25^hi^, and CD127^hi^ expression. As can be seen, the expression is greatly enhanced by stimulation for 5 days with IL-2 and TGF-β.

Figure S2 Stimulation responses of B cells, age distribution of transitional cell fractions, and B cell responses in selective IgA deficiency (IgAD) and healthy controls (HC) to T cell-dependent and T cell-independent stimuli. **(A)** IgA production as measured by enzyme-linked immunosorbent assay (ELISA) from HC isolated B cells after different stimuli. CD40 ligand (CD40L), anti IgM, IL-10, IL-2, IL-4, and CpG. Each bar represents five independent individuals tested in two different experiments. **(B)** Age distribution of transitional B cells in IgAD and HC. The line shows a linear regression for transitional B cell fractions compared to age, no correlation is seen, *R*^2^ = 0.01923, *p* = 0.5. **(C)** IgA production as measured by ELISA and **(D)** Expression following stimulation in HC but not in IgAD individuals CD40L, anti IgM with or without IL-10, and CpG with and without IL-10 in IgAD and HC.

## Abbreviations

AimV, Aim V serum-free medium; ASC, antibody secreting cells; ANA, antinuclear antibodies; BCL6, B-cell lymphoma 6 protein; CCP, cyclic citrulinated peptides; CD40L, CD40 ligand, CD154 is an alternative name; CFSE, fluorescent 5,6-carboxyfluorescein succinimidyl ester; CpG, CpG Oligodeoxynucleotide, TLR9 agonist.; EBV-B, Epstein–Barr transformed B cells; ELISA, enzyme-linked immunosorbent assay; ENA, extractable nuclear antigens; FoxP3, forkhead box P3; GATA3, trans-acting T-cell-specific transcription factor GATA-3; ICOS, inducible T-cell costimulator; IgAD, selective IgA deficiency; IMDM, Iscove’s modified Dulbecco’s medium; IFNγ, interferon gamma; n/iTreg, natural/induced T regulatory cells; PB, peripheral blood; PBMC, peripheral blood mononuclear cells; PD1, programmed cell death protein 1; RF, Rheumatoid factor; RORγT, Retineic-acid-receptor-related orphan nuclear receptor gamma; SEA, SEB, SEE, Staphylococcal enterotoxins; SLE, systemic lupus erythematosus; Tbet, T-box transcription factor TBX21; TFH, T follicular helper cell; TGF-β1, transforming growth factor beta 1; TGF-β1, transforming growth factor beta 1; TH1/2/17/22, T helper cell 1/2/17/22, TLR9, toll-like receptor 9.
