# Supplementary material for: Transitional B Cells and TLR9 Responses Are Defective in Selective IgA Deficiency
Source: Front Immunol. 2018 Apr 27;9:909. doi: 10.3389/fimmu.2018.00909 (PMC5934527; doi:10.3389/fimmu.2018.00909)

Supplement figure 1: Gating hierarchy to identify subsets of T and B lymphocytes.

The figure shows the gating strategy described in the methods for T and B cells. (A) Cells were first identified based on forward scatter (FSC) and side scatter (SSC) and doublets excluded by forward area (FA) and forward height properties (FH) (B) B cells where identified based on their expression of CD19 while excluding CD14+ and CD3+ positive cells and dead cells. Then a gating was done based on CD19 and CD27 to get CD19 low CD27hi B cells. B cells where further analysed based on their different expression of CD20 and CD27 for naïve, memory and Plasmablasts/Plasma cells; CD24 and CD38 for transitional B cells, IgD and CD27 for class switched memory B cells. A gating is shown for HLADR expression on plasmablasts/plasma cells and for the expression of IgA and IgG in both Healthy controls (HC) and IgAD individuals on plasmablasts/plasma cells. (C) T cells where identified based on their expression of CD3 and furthermore evaluated for their expression of CD4 and CD8. The subsets where subsequently analysed for their expression of CD28 vs CD27 and CCD62L vs CCR7. nTregs and iTregs cells where assessed based on their expression of CD4, CD127 negativity, CD25high and CD127high expression. As can be seen the expression is greatly enhanced by stimulation for 5 days with IL-2 and TGF- β.

Supplement figure 2: Stimulation responses of B cells, age distribution of transitional cell fractions and B cell responses in IgAD and HC to T cell dependent and T cell independent stimuli.

(A) IgA production as measured by ELISA from healthy control isolated B cells after different stimuli. CD40L, anti IgM, IL-10, IL-2, IL-4 and CpG. Each bar represents 5 independent individuals tested in two different experiments. (B) Age distribution of transitional B cells in IgAD and HC. The line shows a linear regression for transitional B cell fractions compared to age, no correlation is seen, R2 = 0,01923, p= 0.5. (C) IgA production as measured by ELISA and (D) Expression following stimulation in healthy controls but not in IgAD individuals CD40L, anti IgM with or without IL-10 and CpG with and without IL-10 in IgAD and HC.

Supplement figure 1

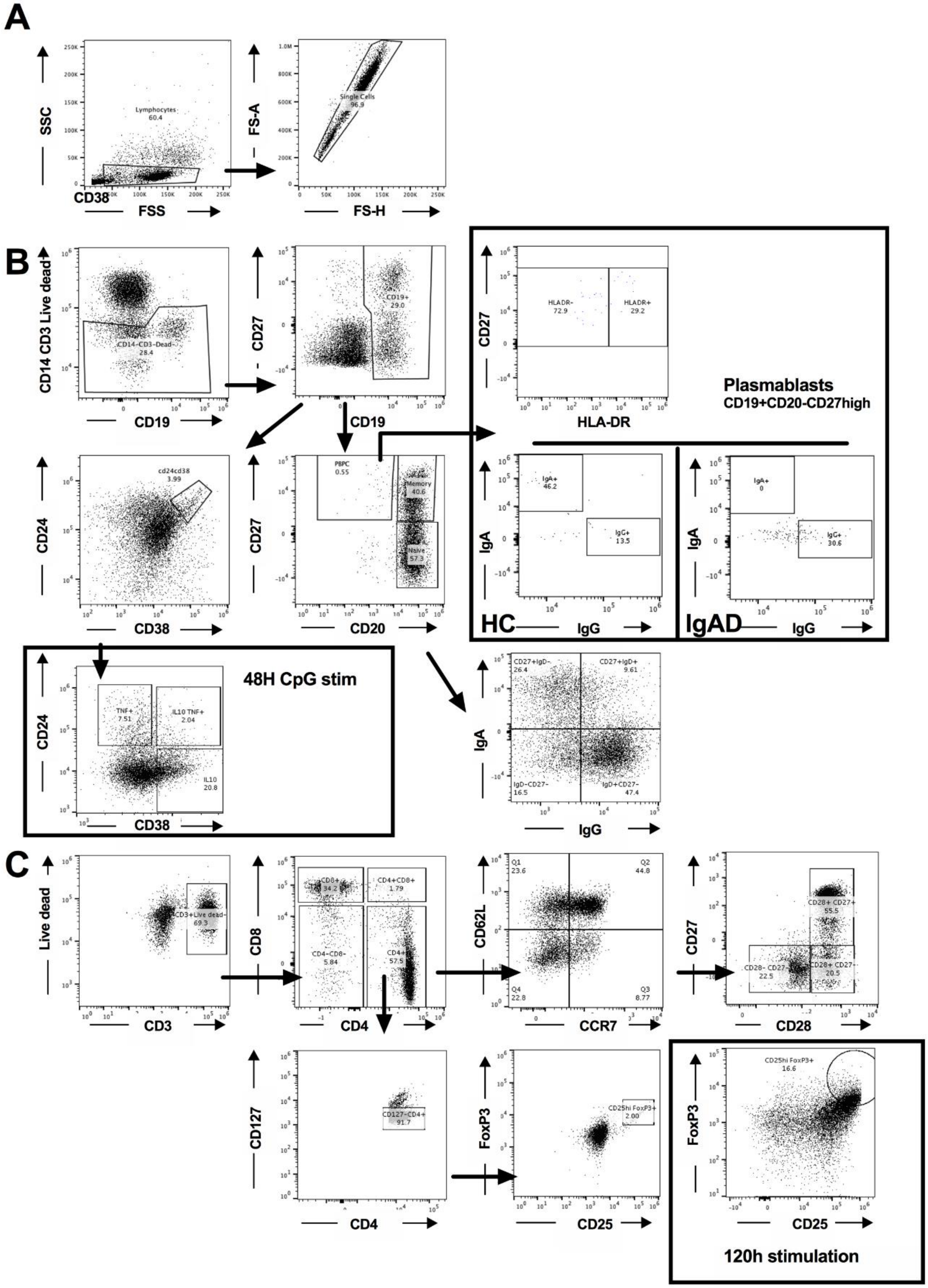

Supplement figure 2

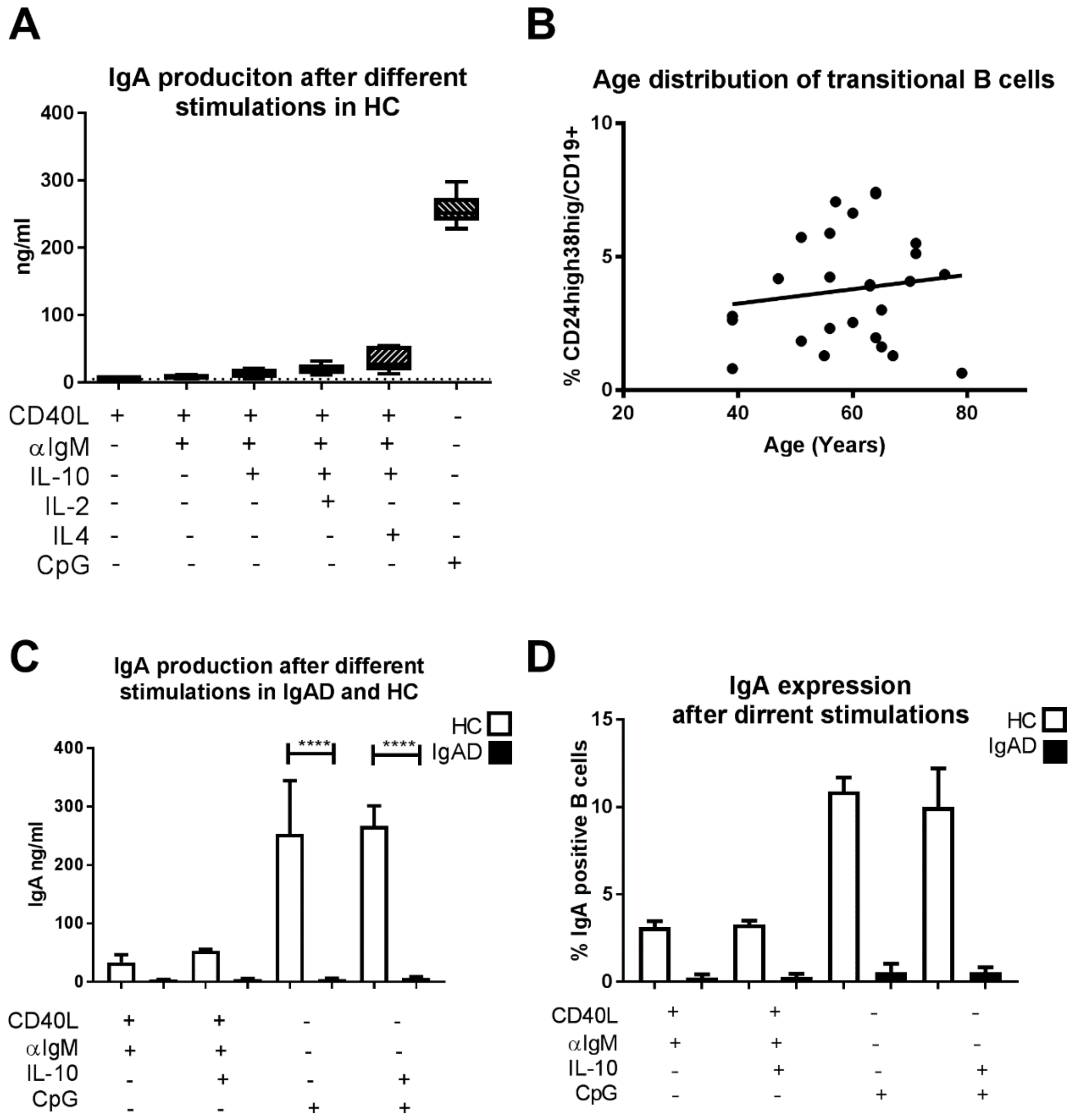

Supplement: Figure S1 — Gating hierarchy to identify subsets of T and B lymphocytes. The figure shows the gating strategy described in the methods for T and B cells. (A) Cells were first identified based on forward scatter and side scatter and doublets excluded by forward area and forward height properties. (B) B cells were identified based on their expression of CD19, while excluding CD14+ and CD3+ positive cells and dead cells. Then a gating was done based on CD19 and CD27 to get CD19loCD27hi B cells. B cells were further analyzed based on their different expression of CD20 and CD27 for naïve, memory, and plasmablasts/plasma cells; CD24 and CD38 for transitional B cells, IgD, and CD27 for class-switched memory B cells. A gating is shown for HLADR expression on plasmablasts/plasma cells and for the expression of IgA and IgG in both healthy controls (HC) and IgAD individuals on plasmablasts/plasma cells. (C) T cells were identified based on their expression of CD3 and furthermore evaluated for their expression of CD4 and CD8. The subsets were subsequently analyzed for their expression of CD28 vs. CD27 and CCD62L vs. CCR7. Natural Tregs and induced Tregs cells were assessed based on their expression of CD4, CD127 negativity, CD25hi, and CD127hi expression. As can be seen, the expression is greatly enhanced by stimulation for 5 days with IL-2 and TGF-β. [file Presentation_1.PDF]
